# Developing a Basic Medical Insurance Statement in Chinese Underdeveloped Areas

**Published:** 2018-04

**Authors:** Hongpu HU, Yanli XU, Qingkun CHEN, Juan LI, Chenliu YANG, Yanli WAN

**Affiliations:** 1. Institute of Medical Information, Chinese Academy of Medical Sciences, Beijing, China; 2. Medical College of Hebei Engineering University, Handan, Hebei, China

**Keywords:** Underdeveloped areas, Rural basic medical insurance system, Development experiences

## Abstract

**Background:**

We presented the running state of rural basic medical insurance system in Henan and discussed the enforcements and development experiences of underdeveloped areas. We provided data evidence to support the improvement and development of a basic rural medical insurance system.

**Methods:**

We selected Henan Province, China as a sample, using the method of cluster sampling, from policy documents published in the national and provincial level of the new rural cooperative medical policy and work documents, data from 2004 to 2014, the National Health Statistical Yearbook of health statistics yearbook of Henan Province and relevant statistical data of the province.

**Results:**

The new rural cooperative policy has covered the whole population in Henan Province. The number of individual received benefits is increasing. In 2013, the number of persons counted has reached to 270 million, funds raised and expenditures reached 38.5 billion and 26 billion, respectively. The operational task force has been developed rapidly. In 2013, on average each staff managed the cases for 16.4 thousand rural residents.

**Conclusion:**

The major implementation and development experience from the new rural cooperative policy of Henan province include: education of related knowledge, optimization of compensation plan, development of operational system and framework, improvement of management rules, reinforcement of information system development and financial supervision and increment of investment in rural medical healthcare.

## Introduction

As an economically unbalanced agricultural country, China still has realistic problems that limit the developments to become a well-off society and towards modernization, such as excess rural population and the following three rural issues including agriculture, rural areas and farmers (1). Therefore the physical and mental health of farmers and their satisfaction of rural medical services are especially important to the future of medical developments. In the meantime, establishing a high quality rural basic medical insurance system, providing superior medical health services to farmers and reducing economic pressures of farmers are important to narrow the urban-rural gap, build new socialistic villages and maintain social stability. The rural cooperative medical care system in China has been developed for more than 60 years, and it makes it effective to provide appropriate medical health services to farmers with only a small amount of healthcare co-pays from farmers at low income level. To keep up with the modernization of the society and resolve the problems for rural residents to seek medical advice, the Central Committee of the Communist Party of China (CPC) and the State Council promulgated “the Decision on Rural Health Works Enhancement” in October 2002, and it indicated the purpose of the new rural cooperative medical system (NCMS). In the second half year of 2003, the implementation of NCMS officially started. In ten years, the government has improved rural basic medical treatment, established a medical system, and solved the difficulty and high cost for farmers to receive quality medical treatment, and NCMS has achieved major success.

Currently, the economic development of China is imbalanced, the eastern region of China experiences rapid development while the middle and western regions are being developed sluggishly. A good economy should guarantee the enforcement of rural medical insurance system, however the low economy status of underdeveloped areas limits the coverage and compensation of rural medical insurance system (2). Meanwhile, the large rural population and the increasing medical service need swill also propel rural medical insurance system to become a significant basic policy including active exploration and constant improvement. This study applied cluster sampling method to data from Henan province which has an under-developed economic status to present the current state of rural basic medical insurance system, and discuss the experiences about how underdeveloped areas establish rural basic medical insurance system. Finally, we provided scientific evidence regarding rural basic medical insurance system improvement and enforcement.

## Methods

This study selected Henan Province as a sample, using the method of cluster sampling, from policy documents published in the national and provincial level of the new rural cooperative medical policy and work documents, data from 2004 to 2014, the National Health Statistical Yearbook of health statistics yearbook of Henan province and relevant statistical data of Henan Province. The main indicators of policy effectiveness evaluation include farmers’ participation Status, benefit situation of NCMS Farmers, financing situation of NCMS, development of NCMS Agencies, defined as:

The participation of farmers = the number of participating farmers in the province / the total number of farmers in the province ×100%;
The benefit of participating farmers = the number of compensation farmers in the province / the number of participating farmers in the province ×100%.

Henan Province is located in the middle of China, near the middle and lower segment of the Yellow River, southwest of Huang-Huai Plain, next to 6 provinces including Shandong, Anhui, Shanxi, Hubei, Shanxi and Hebei. Henan has 18 prefecture-level cities: Zhengzhou, Kaifeng, Luoyang, Pingdingshan, Anyang, Hebi, Xinxiang, Jiaozuo, Puyang, Xuchang, Luohe, Sanmenxia, Nanyang, Shangqiu, Zhoukou, Zhumadian, Xinyang, Jiyuan, abuts 5 cities: Gongyi city, Xiangcheng city, Yongcheng city, Gushi city, Dengzhou city, and a total of 103 counties. Henan has a large total population and a large agricultural population; in 2010, the population in Henan took 7.01% of the total population of China. While China’s rural population was 8.57% of the total rural population of China, Henan’s rural population (57,540 thousand) was 61.2% of the total population (94,050 thousand) of Henan Province (2). Comparing to other provinces in China, Henan is at a lower economic development stage and has a larger population, lower cultural education level, less cultural quality, serious outflow of higher educated residents, unsubstantial economic foundation, insufficient long-term investment and higher proportion of poverty counties, which all limit the developmental speed of Henan.

In this study, the SPSS software (Chicago, IL, USA) was used to make a descriptive statistical analysis of the above indexes by using the average number, median, rate and so on.

## Results

### Development History of Henan Rural Basic Medical Insurance System

Henan is a large agricultural province. The total rural cooperative population of farmers is 81,190,000. Properly solving most rural civilians’ medical insurance problems is the most key priority to people in the province. After the founding of the People’s Republic of China, Henan rural basic medical insurance system experienced multiple stages from sprout, initiation, development, climax, recession, recover rebuilding, second time cooperative medical treatment to the NCMS phase, and it went with constant exploration (3).

The initial stage of country establishment (1949–1965) is the sprout of Henan rural basic medical insurance system. In 1955, Henan health department announced “Joint Medical Institutions Regulation”, it was the policy basis of the province’s cooperative medical treatment. During the period of 1965 to 1980, Henan rural cooperative medical treatment started to reach a new level. Henan took a series of actions to focus on village works, including training medical health teams, organizing urban medical health workers to go to villages, transferring medicine, medical and instruments to villages, and these actions produced many achievements (4). From 1980 to 1994, cooperative medical stations successively dismissed, and transformed to various forms of healthcare providers. Subsequently, the large scale household-responsibility system implementation weakened collective force, cut off the funding sources of rural cooperative medical treatment system, and Henan rural cooperative medical treatment system was on the wane (5). After 1990s, the Chinese government advocated for recovery and rebuilt rural cooperative medical treatment system. In 1994, building from the base of the cooperative medical treatment under low-level organization of people’s communes throughout the country, Henan provincial government and the health department learned from the experiences of 1960s and 1970s to implement rural cooperative medical treatment innovation, but the coverage was still lower than the level of 1970s.

On the basis of the traditional cooperative medical treatment system, in October 2002, CPC central committee, the state council announced “The Decision of Rural Health Works Enhancement”, and clearly indicated steps to build NCMS mainly for severe diseases. In September 2003, Henan Province responded to the call of CPC central committee to start NCMS implementation. NCMS has innovation and development on planning government responsibilities and insurance contents. NCMS is farmers’ medical treatment mutual assistance system which has organization, guidance and support from government, voluntary participation of farmers, financing from individual participants, collectivity and government. Meanwhile, it mainly includes severe disease plan. NCMS usually sets county as the unit of plan as a whole, and it is mainly used as subsidy of large medical treatment fees or hospital medical treatment fees. NCMS, mainly including severe diseases plan as a whole, is a health service institution which match with the requirements of socialist market economic system and the situation of rural social and economic development. In 2008, Henan achieved the complete coverage of NCMS, there were a total of 72,490,000 farmers joined NCMS, and the participation rate rose from 75.58% of first pilot counties in 2003 to 92% (6); then the participation rate went up to 99% in 2013.

### Rural Basic Medical Insurance System Operation Situation in Henan Province

From sprout initial stage to recession dormancy, from recover rebuilding to the rise of NCMS, after difficulties and deeply explorations, Henan has already established NCMS extensive coverage and reached a new situation that farmers physical and mental health care can be covered. According to the national unifying deployment, Henan activated NCMS experimental unit in 2003. Henan provincial government began to enhance financing standards step by step, increased governmental subsidy level, reduced hospitalization expenses starting line, improved hospitalization fees reimbursement ratio and blockade line, and decreased the economic burden of medical treatment. In September 2003, Henan selected Tangyin, Gushi, Lushi, Xiayi, Jiaxian, Taiqian and other 19 counties as experimental units, and started to implement NCMS. Until July 2004, 25 experimental units in Henan Province had 11,971,000 farmers join NCMS, the participation rate is 78.09%, and total financing is 324,441,000 Yuan (7). Until July 1st, Henan expended medical treatment funds 129,192,000 Yuan; 8,207,000 farmers got cooperative medical treatment subsidies, experimental units ran smoothly and welcomed by the public. In 2006, Henan increased experimental units to 67 counties and cities, farmers participation rate is 81% (8). In 2008, Henan achieved full coverage of NCMS, all 157 counties and cities built NCMS, and financing level increased from an initial personal average of 30 Yuan to a personal average of 100 Yuan. Henan NCMS participation population increased from 11,585.800 people of experimental units in 2003 to 81,190,000 people in 2013, participation rate was increased up to 99%.

### Farmers Participation Status

Since the implementation of NCMS, farmers’ participation rapidly increased. Henan selected 25 counties (cities) as experimental units to launch NCMS in 2003; until 2004, Henan NCMS participation population was 11,585,800 people, participation rate was 75.72%. In 2006, the experimental counties (cities) expanded to 65; and the experimental units further added to 143 in 2007, NCMS participation population increased to 59,386,600 people, participation rate went up to 86.87%. Up to 2013, Henan NCMS counties (cities, districts) were 157, NCMS participation population was 81,190,000 people, participation rate was 99%, ranked among the top nationwide (Table 1).

**Table 1:** 2004–2013 Farmers Participation Status in Henan Province

***Year***	***NCMS Participants (thousand)***	***Participation Rate (%)***
2004	11585.8	75.72
2005	11185.6	73.10
2006	32183.5	80.92
2007	59386.6	86.87
2008	72798.4	91.88
2009	74880.0	94.35
2010	76514.8	96.75
2011	78044.6	96.85
2012	79650.0	98.00
2013	81190.0	99.00

### Benefit Situation of NCMS Farmers

From 2004 to 2013, the beneficial NCMS farmers were increasing. Until 2013, beneficial NCMS farmers were 27,128,000 person-times and increased to 25,478,000 person-times from the beginning 1,651 thousand person-times in 2004 (a 16-fold increment). Out-patient compensation occupied major proportion (85%). Significant changes happened every year, but the general was on the rise (Table 2).

**Table 2:** 2004–2013 Benefit Status of NCMS Farmers in Henan Province

***Year***	***Benefit People (thousand)***	***In-hospital Compensation***		***Outpatient Compensation***		***Others***	
		Compensation people (thousand)	Rate (%)	Compensation people (thousand)	Rate (%)	Compensation people (thousand)	Rate (%)
2004	16509.87	464.544	2.81	15185.02	91.98	860.305	5.21
2005	7551.944	561.512	7.44	6435.676	85.22	554.756	7.34
2006	19070	1538.133	8.06	16022.04	84.02	1509.832	7.92
2007	31305.49	3637.702	11.62	26651.12	85.13	1016.661	3.25
2008	36498.31	5031.171	13.78	30434.93	83.39	1032.211	2.83
2009	49506.79	5252.853	10.61	43204.64	87.27	1049.288	2.12
2010	115445.7	5960.8	5.16	108302.8	93.81	1182.1	1.03
2011	982957.4	5975.758	6.08	891490.8	90.69	85490.78	3.23
2012	197665.5	7,98	3.79	178401.9	90.25	11765.5	5.96
2013	271286.1	8342.8	3.08	204579.1	75.41	2653.1	0.97

### Financing Situation of NCMS

From 2004 to 2013, the total amount of NCMS financing was increasing. The proportions of central finance and local finance had a stable variation trend; the proportion of total amount of farmers’ financing was constantly reducing, the financing rate changed from 24.72% of 2005 to 9.32% of 2008 (a 15.4% dropping), and it started to go up in 2009 (Table 3).

**Table 3:** 2004–2013 Financing status of NCMS in Henan Province

***Year***	***Total Funds (thousand Yuan)***	***Central Finance Subsidies***		***Local Finance Subsidies***		***Farmers’ Financing***	
		Total amount (thousand Yuan)	Rate (%)	Total amount (thousand Yuan)	Rate (%)	Total amount (thousand Yuan)	Rate (%)
2004	466200.8	170759	36.63	183486.3	39.36	111835.5	23.99
2005	452550.7	82910	18.32	113765.8	25.14	111856.1	24.72
2006	1507376	515535.8	34.20	656737.9	43.57	321935.8	21.36
2007	3660489	1256170	34.32	1199584	32.77	594128.8	16.23
2008	7840714	2808740	35.82	2938495	37.48	730894.6	9.32
2009	9870518	3062952	31.03	3018380	30.58	1513576	15.33
2010	14266750	4591810	32.19	4607220	32.29	2298350	16.11
2011	22694790	8428820	37.14	7215470	31.79	2351290	10.36
2012	31825570	10513950	33.04	8633180	27.13	3990890	12.54
2013	38534130	13949000	36.19	9377000	24.3	4895000	12.7

### Funding Usage of NCMS

From 2004 to 2013, the total expenses of NCMS funds were elevated, increased to 25,991 million Yuan in 2013 from 330 million Yuan in 2004. Among the annual fund service conditions of NCMS, the funds of NCMS were mainly comprised of in-hospital compensation expenses, and the proportion of total in-hospital compensation expenses was decreasing in trend yearly. The proportion of total in-hospital compensation expenses reduced from 86.8% in 2008 to 78.94% in 2010 (a 7.86% decrease); the proportion of out-patient compensation expenses was kept under about 25% except year 2004 (Table 4).

**Table 4:** Funding Usage of NCMS

***Year***	***Total Fund Expenses (thousand Yuan)***	***In-hospital Compensation***		***Outpatient Compensation***		***Others***	
		Total amount (thousand yuan)	Rate (%)	Total amount (thousand yuan)	Rate (%)	Total amount (thousand yuan)	Rate (%)
2004	326,677.6	199,026.2	60.92	123,647.1	37.85	4,004.3	1.23
2005	297,781.8	214,057	71.88	73,055.9	24.53	10,668.9	3.59
2006	966,835	709,574.8	73.39	229,551.1	23.74	27,709.1	2.87
2007	2,445,135	1,951,032	79.79	463,372.3	18.95	30,730.4	1.26
2008	5,523,329	4,794,306	86.8	621,498.5	11.25	107,524	1.95
2009	7,036,631	5,781,698	82.17	1,125,815	16	129,117.4	1.83
2010	9,956,318	7,859,069	78.94	1,919,243	19.28	178,006.6	1.78
2011	13,173,160	10,458,280	79.39	2,444,672	18.56	270,206.4	2.05
2012	21,945,440	17,345,130	79.04	3,994,460	18.20	605,850	2.76
2013	25,991,000	19,538,000	75.17	8,123,000	19.71	1,330,000	5.12

### Development of NCMS Agencies

From 2005 to 2013, the number of NCMS authorized employees increased rapidly, and 4951 employees were hired in 2013 while it had only 243 employees in 2005. On average, each employee supervised 47.5 thousand NCMS farmers in 2004, while each employee managed 16.4 thousand NCMS farmers in 2013. The incomes of NCMS agencies were increasing, annual expenses and incomes were almost matched; they increased rapidly from 2008 to 2009 and the increasing speed started to reduce since 2010 (Table 5).

**Table 5:** Development of NCMS Agencies

***Year***	***Employees***	***Average Management of NCMS farmers (thousand Yuan)***	***Annual Income (thousand Yuan)***	***Annual Expenses (thousand Yuan)***
2004	244	47.5	7,021.4	7,813.4
2005	243	46	5,412.3	6,465.6
2006	1006	32	29,245.9	30,909.9
2007	1741	34.1	46,185	52,245.6
2008	3051	23.9	52,625.6	59,714.2
2009	4458	16.8	70,078.6	137,876.3
2010	4907	15.6	93,939	93,638.5
2011	4984	15.7	109,762.9	109,205.1
2012	5016	15.9	120,300	123,700
2013	4951	16.4	135,510	132,280

## Discussion

### Popularizing relevant knowledge and improving the peasant’s willingness of NCMS participation.

Since NCMS started to implement in Henan Province, the number of NCMS participants and participation rate had largely increased, and almost achieved full coverage. At the beginning of NCMS enforcement in 2004, the total participants of 18 counties (cities) were 11,585,800, participation rate was 75.72%; in 2005, the total participants were 11,185,600, participation rate was 73.1%. At this stage, because NCMS just has been implemented, farmers did not sufficiently understand and trust this new system, so participation rate was low at that time. With regard to this problem, different local government agencies took several approaches to popularize the new system, such as newspapers, radio broadcast, television and other media; moreover (9), they popularized health care and NCMS relevant knowledge to farmers, tried to enhance farmers’ awareness to self-health care, and encouraged farmers to take part in cooperative medical treatment. In addition, all levels of governments were working on improving relative NCMS policies, optimizing compensation program, reducing farmers’ burdens, and making farmers voluntarily participate in NCMS. With the enforcements of various relevant measures, farmers had more understandings of NCMS and better participation. NCMS participants went up to 32,183,500 in 2006, participation rate was 80.92%. In 2013, NCMS participants increased to 84,490,000 in Henan province, participation rate was 96.85%, achieving full coverage of NCMS.

### Optimizing compensation program and enhancing insurance level

Since NCMS started to implement in Henan province, according to local situation and the principles that expenditure should be determined by revenue while maintaining balance of payments with some surplus, Henan built the compensation program that severe diseases were major part of coverage of the program and took care of out-patient disease at same time; meanwhile, Henan also enhanced the governmental subsidy level step by step. From 2003 to 2006, the governmental personal average subsidy was 20 Yuan, personal payment was 10 Yuan, and total financing was 30 Yuan. In 2012, the fifth increment of governmental personal subsidy, the average subsidy was 240 Yuan, it included the central government subsidy of 132 Yuan, local financial aid 10 Yuan and personal payment of 50 Yuan, total financing was 290 Yuan. At the same time with financing increment, Henan reduced starting line for 6 times, increased the maximum pay line and the proportion of medical treatment compensation and solved expensive problems of farmers’ medical treatment step by step. The maximum pay line increased from original 5000 Yuan to 150,000 Yuan in 2012, and it was 27 times greater than Henan rural civilians’ annual incomes, ranked first in the country. To improve the farmers’ economic risk abilities of severe diseases prevention, from 2004 to 2013, Henan’s total NCMS expenses increased, in-hospital compensation increased from 199 million Yuan to 25,991 million Yuan, in-hospital compensation was also on the rise; meantime, Henan provincial government increased reimbursement ratio to 80% when the in-hospital costs were up to 60,000 Yuan onetime, increased reimbursement ratio to 90% when the in-hospital costs were up to 100,000 Yuan one-time, and it reduced the farmers’ economic burdens of disease to a certain extent (10).

### Constructing basic operational framework and improving management regulation

After 9 years of developments and explorations of NCMS, Henan had built a comprehensive rules and regulations, expanded management teams and increased office expenses. All districts of Henan constructed the organizational system and management operation mechanism which was led by local government, managed by the health department, cooperated with relevant departments, operated by insurance agencies, served by medical institutions and participated with farmers. They reset NCMS management institutions from villages to counties and the administrative department above county level and scaled up the management level (11). The number of NCMS agencies’ authorized employees increased rapidly, and it hired 4951 employees in 2013 while it had 243 employees in 2005. Annual expenses of NCMS agencies increased from 7,813.4 thousand Yuan to 132,280 thousand Yuan, and office expenses also went up. At the same time, Henan actively explored the new model through the combination of NCMS and commercial insurance. Xinxiang city and Luoyang city built “Xinxiang Model” and “Luoyang Model” during these years and got effective impacts. In Jul. 2011, Zhengzhou city started setting experimental units in administrative counties (cities, districts). This new model combined NCMS and commercial insurance used purchase service mode, entrusted commercial insurance company to manage the verification, settlement, payment and other services of medical costs, paid handling fees to commercial insurance company based on several criterions, and established the NCMS management operation mechanism that led by party committee government and supervised by the health department and handled by commercial insurance companies, which fully used resources organization and scientific management experiences of commercial insurance companies and supplemented funds to NCMS, eventually enhanced farmers’ benefits.

### Constructing NCMS provincial information platform and implementing real-time settlement

To simplify the reimbursement program, manage the reimbursement process and enhance work efficiency, Henan started to build NCMS information platform since 2005, completed the NCMS provincial information platform construction in August 2010, finished NCMS information platform and connected with other 150 provincial counties (districts), implemented data sharing between province level and county level and completed connection with the county platform. The instant reimbursement system worked as a main part of the provincial NCMS information system, achieved connections among cities, counties and villages in the end of 2008, opened up 158 counties’ direct subsidy works, and it also completed the connections between NCMS farmers and provincial directed hospitals. In 2011, Henan province implemented trans-regional instant reimbursement, completed connections of NCMS information platforms and laid the foundation of verification and immediate settlement of trans-provincial medical costs (12). In January 2012, Henan Province built NCMS directed medical institutions for the farmers who went out of hometown for work allowing them to join NCMS and get medical treatment and reimbursement in their work places. Currently, different districts of Henan had already constructed almost 100 farmers direct-access hospitals in Beijing, Jiangsu, Shanxi, Shandong, Fujian, other provinces and Guangzhou, Shenzhen, Zhengzhou and other cities. In the process of information construction, Henan Province insisted unified software development, hardware construction, funds usage, employee training and daily supervision, which fully produced the best possible achievements of funds, technologies, employees, improved relevant rules and standards, step by step. Finally presented key points and produced effective impacts (13).

### Reinforcing supervision and ensuring funding operation security

The long-term developments of NCMS would not go well without effective supervision system for medical treatment service and funding operation management. Henan took a series actions to improve supervision, such as constructing employees’ recruiting and dismissing regulations, agreement management regulations, supervision and inspection regulations, analysis report regulations and accountability system. To ensure the NCMS funds security and being effectively use, all-levels of governments actively improved NCMS funds finance and accounting system and created system so that the deposit of fiscal special account and income-expenses management were running in parallel (14). They also guaranteed the appropriation method for NCMS financial subsidy fund application, constructed NCMS fund special auditing and financial supervision censorship, established publishing system of NCMS funds’ income and expenses, service conditions, large cost subsidies and built overdraft risk warning and notification systems.

### Increasing rural medical treatment and public health input and improving fairness and universality of health services

Good medical and health conditions were the foundation of NCMS operation. Since NCMS implementation, Henan has gradually increased the villages and towns’ health resources investment, enhanced hospital developments in villages and towns, improved rural health resources usages, reduced medical burdens of big hospitals in city and effectively distribute patients. According to China health statistic yearbook, the average health department employees of each Henan village were 3.17 people in 2010, exceeded 1 person than country average level (2.17 people). Rural areas’ clinics had 1.78 employees for every 1,000 people. In 2011, Henan province got central investment of 1.56 billion Yuan, plus local support of 0.99 billion Yuan, which supported 24 county level hospitals’ extension and innovation, 78 central villages and towns’ health centers, 27 cities and counties’ mental health institutions, 51 county level emergency command centers, 130 county level health supervision institutions and 7 general practitioner clinical training bases. Henan also achieved the standard that each county had at least 1 county level hospital and 3 to 5 central villages and towns’ health centers. Provincial finance arranged directed funding of 100 million Yuan to rebuild 10,000 health centers. Provincial finance arranged 60,330,000 Yuan to subsidize 2027 employees to continue advanced education in the future, recruited in 2,600 medical college graduates and trained 2,089 village doctors. To provide convenience access for NCMS farmers’ to nearby medical treatments and nearby reimbursement, Henan province listed eligible village health centers as NCMS direct affiliated medical institutions. The general charge for medical treatments and medicines were eligible for NCMS payment. To insure the income of village doctors, Henan province provided compensation based on the number of services and qualities using multiple ways to village health centers which belonged to basic medicine system and was part of NCMS out-patient service plan. This included basic public health services’ subsidies, basic medical services compensation and 5,000 Yuan basic city medicine system direct subsidy to cities and villages’ health centers for each 1,000 agricultural register people every year. The doctors who worked as village doctors for more than 10 years, retired because of age, no longer worked as doctors, are provided 300 Yuan subsidies per person per month (15).

Henan Province was among underdeveloped areas in China, however, it took farmers’ basic medical insurance system as a fundamentally important policy, actively explored, constantly provided health policy and management improvement, which provided valuable experience regarding the implementation of farmers’ basic medical insurance system in other areas.

## Conclusion

The major implementation and development experience from the new rural cooperative policy of Henan providence include: education of related knowledge, optimization of compensation plan, development of operational system and framework, improvement of management rules, reinforcement of information system development and financial supervision and increment of investment in rural medical healthcare.

## Ethical considerations

Ethical issues (Including plagiarism, informed consent, misconduct, data fabrication and/or falsification, double publication and/or submission, redundancy, etc.) have been completely observed by the authors.
